# Negotiating Gender in Everyday Life: Toward a Conceptual Model of Gender Dysphoria in Adolescents

**DOI:** 10.1007/s10508-021-02024-6

**Published:** 2021-10-29

**Authors:** Reidar Schei Jessen, Anne Wæhre, Linda David, Erik Stänicke

**Affiliations:** 1 Division of Clinical Neuroscience, Oslo University Hospital, Kirkeveien 166, 0450 Oslo, Norway; 2 Division of Pediatric and Adolescent Medicine, Oslo University Hospital, Oslo, Norway; 3 Department of Psychology, University of Oslo, Oslo, Norway

**Keywords:** Gender dysphoria, Gender affirmative care, Transgender, Gender non-conforming youth, Phenomenology, Gender identity

## Abstract

A growing number of adolescents are seeking medical care to alleviate gender dysphoria (GD). This qualitative study explored the subjective experiences of GD among help-seeking transgender and gender nonconforming (TGNC) youth in order to develop a more nuanced conceptualization of the phenomenon. Fifteen life-mode interviews were conducted with newly referred youth between the ages of 13 and 19. All participants were assigned female at birth. The data were analyzed using thematic analysis. The participants targeted five major themes that characterize GD: (1) Bodily sensations were constant reminders of GD throughout the day, (2) emotional memories from the past of being different and outside triggered GD, (3) the process of coming out was a transformative experience that changed how the participants understood themselves, (4) GD both increased and decreased in relation to others, (5) everyday life required careful negotiation to feel whole without developing new forms of GD. Based on the results, we suggest a more conceptually nuanced model of GD, one which accounts for how bodily sensations and emotional memories from the past were sources that elicited GD. The sources were mediated through the process of coming out and relating to others, and this resulted in the negotiation of GD today. The conceptual model suggested in the present study could ideally shed light on preexisting knowledge on TGNC youth struggling with GD. In addition, an improved understanding of GD could ideally help clinicians when addressing individual treatment needs.

## Introduction

Transgender and gender non-conforming (TGNC) refers to individuals that experience a degree of incongruence between their internal sense of gender (gender identity) and the sex assigned at birth (Winter et al., 2016). Today, the diagnoses of gender dysphoria (GD) in the fifth edition of the *Diagnostic and Statistical Manual of Mental Disorders* (American Psychiatric Association, 2013) and gender incongruence in the 11th revision of the *International Classification of Diseases* (World Health Organization, 2019) are used to classify treatment needs among TGNC individuals. These diagnoses refer to the distress that arises from the mismatch between gender identity and assigned sex at birth (Butler et al., 2018). The diagnostic classifications of (trans)gender identity and the medical perspectives of health care for TGNC individuals are an area long characterized by misconceptions, controversy, and a lack of knowledge (Drescher et al., 2012; Winter et al., 2016). Today, the psychopathological model of TGNC and GD, which is based on outdated Western medical conceptualizations of gender and sexuality, is no longer in use (Wylie et al., 2016). We are instead encouraged to move toward a model of GD that incorporates current scientific evidence and best practices with the experiences of TGNC people themselves (Drescher et al., 2012).

For some TGNC people, medical treatment aimed to bring the body more in accordance with internally felt sense of gender is necessary to alleviate GD (Butler et al., 2018; de Vries & Cohen-Kettenis, 2012; Drescher et al., 2012). In the global West since the mid-2000s, there has been an increase in the number of teenagers referred to clinics in an effort to alleviate GD through gender-affirming healthcare (de Graaf et al., 2018; Kaltiala et al., 2020a; Zucker et al., 2008). This has created increased attention both within clinics and in public debate regarding what constitutes best treatment for TGNC youth seeking such help (Bell, 2020; Saketopoulou, 2020; Wren, 2019). The first evaluative research of medical treatment for adolescents was promising. It indicated that carefully selected youth without significant psychosocial challenges benefit from puberty suppression followed by hormonal treatment after age 16, in regard to alleviating psychological distress related to GD (Cohen-Kettenis & van Goozen, 1997; de Vries et al., 2014). In parallel with the increase in adolescent referrals over the past few years, there has been a shift in gender proportion—a majority of the youth referred to the gender clinics today are assigned female at birth (Arnoldussen et al., 2019).

Furthermore, we know that TGNC youth with GD as a group suffer more from co-occurring mental health challenges than do their peers (Chodzen et al., 2019; Leibowitz & de Vries, 2016; Olson-Kennedy et al., 2016). Some clinicians and researchers are concerned that for certain TGNC youth, subjective experiences of GD are related to underlying mental health issues or difficulties in adolescent development that do not decrease after medical treatment (Butler et al., 2018; Carmichael et al., 2021; Kaltiala et al., 2020b). Other clinicians and researchers subscribe to the minority stress hypothesis, which posits that that co-occurring mental health challenges among TGNC youth are the result of growing up in environments that are not inclusive of gender non-conformity (Chodzen et al., 2019). Furthermore, it has been hypothesized that co-occurring psychopathology will remit when the body is changed in accordance with gender identity, and the young person is able to pass as the preferred gender (Chodzen et al., 2019; Ehrensaft, 2017). Thus, the current body of knowledge on TGNC youth with GD indicates that they comprise a heterogeneous group with various clinical needs (Janssen et al., 2019).

### Phenomenological Knowledge on GD

Most of the research on TGNC youth and clinical needs has been quantitative, consisting of questionnaires on psychiatric symptoms and livelihood measurements (Giovanardi, 2017; Olson-Kennedy et al., 2016). At the same time, experiential qualitative analyses of individual TGNC youth’s experiences that “give voice” to their perspectives are also needed in order to more fully understand GD among the TGNC adolescents referred to medical treatment (McLeod, 2013). Qualitative research indicates that being able to find relationships that both affirm one’s gender identity and enable gender role casting is important for alleviating GD (Loza et al., 2017; Mullen & Moane, 2013). Levitt and Ippolito (2014) interviewed TGNC adults about their gender identity development. The study identified a common process of growing up closeted and full of self-hatred, followed by increased possibilities of self-exploration and self-acceptance after learning about TGNC narratives. Identity formation was described as a process continuing into adulthood, whereby one learns to balance authenticity with the need to survive discriminatory conditions. The findings resonated with stage models of TGNC identity development, which we discuss further in the section on implications (Bockting, 2014; Devor, 2004).

However, there has been little systematic investigation on phenomenological aspects of GD, namely how GD is understood and experienced by the youth themselves. Steensma et al. (2010) investigated GD qualitatively among youth from a clinical population. The youth interviewed in their study reported that the pubertal development of the body, together with an accentuated social division between boys and girls in school and the first romantic and sexual experiences in early adolescence, increased subjective experiences of GD, thus suggesting a complex interaction between bodily and social factors that contribute to GD. The importance of the body, especially under pubertal changes, in the development of GD was also identified by Katz-Wise et al. (2017), McGuire et al. (2016), and Pollock and Eyre (2010). Furthermore, qualitative studies on TGNC youth have documented the powerful role of forming relations to others in order to explore gender identity (Austin, 2016; Catalpa & McGuire, 2018; McGuire et al., 2016; Pollock & Eyre, 2010; Riggs et al., 2019; Wilson et al., 2005), and the need for cultural recognition of minority gender identities (Bradford et al., 2018; Katz-Wise et al., 2017; McGuire et al., 2016; Salzburg & Davis, 2010).

The subjective and experiential elements of GD are especially important, since the mismatch between internal sense of gender and assigned sex at birth is the target of medical interventions aimed to make the body more in accordance with gender identity (American Psychiatric Association, 2013; World Health Organization, 2019). Within the perspective of phenomenology, the aim is to shed light on how the individual subject experiences a phenomenon in daily life (Vetlesen & Stänicke, 1999). Furthermore, phenomenology shows that everything is experienced “as something.” The aim is therefore to discover the essence of the experience (Dahlberg, 2006). In the present study, the focus is on subjective experiences of GD. An improved conceptual understanding that specifies the relations among clusters of subjective experiences could potentially bridge the gap between the research literature on large group data variables that we have previously accounted for and the individual variance within the group (Olson-Kennedy et al., 2016).

### The Present Study

We aimed to fill the lack of qualitative and phenomenological knowledge in the research literature by conducting an interview study with newly referred youth to the National Treatment Unit for Gender Incongruence at Oslo University Hospital. In order to describe the subjective experiences of GD more in detail, the study was guided by the following research questions:

1. Which experiences do adolescents assigned female at birth target as essential when interviewed about GD in their daily life?
2. How can the results be conceptualized into a model that establishes the connections between the clusters of experiences in order to contribute to the growing body of knowledge on development of GD in adolescence?

Furthermore, the findings from the present study could be useful for obtaining a deeper understanding of what clinical strategies can help these young people and their families explore and handle GD in daily life.

## Method

### Participants

The study was planned by a working group consisting of two researchers with work experience as clinical psychologists (first and last authors), in addition to one child and adolescent psychiatrist (third author) and one clinical psychologist (second author). The second and the third author were working at the National Treatment Unit for Gender Incongruence at the time of the study. In addition, a reference group consisting of representatives from one patient organization and two LGBTQ-identifying organizations guided the entire process. The representatives from the reference group gave feedback on the interview guideline and the interview process throughout the data collection in order to ensure that relevant aspects were covered. They also gave feedback on the coding process and the development of themes, as well as the writing-up of the present study.

The aim was to recruit a representative sample of newly referred adolescents seeking medical treatment for GD at at the National Treatment Unit for Gender Incongruence. In the recruitment process, we strived to ensure a diverse background among the participants in relation to gender identity, birth assigned sex, and age. Common to all 15 participants was that none of them suffered from psychosis or severe suicidal behavior. In total, 15 newly referred patients assigned female at birth between the ages of 13 and 19 were recruited. The mean age of the participants was 16. All participants but one identified as male at the time of the interview. Nine participants had not received any medical treatment at all; six had received medical treatment from other health personnel before entering the national treatment service for gender incongruence; three of them had just started with testosterone hormonal treatment, and one had received puberty suppression medication. In addition, two had recently been prescribed birth control pills by their primary physician in order to stop menstruation. Although all participants had been newly referred to the clinic, it appeared that some had taken measures to seek medical treatment to alleviate GD from physicians working in primary healthcare before being referred to the national treatment unit.

### Measures

Semi-structured interviews were used to explore subjective experiences of GD among TGNC youth, because this approach offers an opportunity to balance openness toward individual diversity with a focus on the overarching research question (Kvale & Brinkmann, 2009). A qualitative approach was chosen, because it was deemed best suited for investigating phenomenological and subjective aspects of a certain phenomenon. All interviews were conducted by the first author. In the first part of the interview, participants were encouraged to describe their childhood, and why they had been referred to the gender clinic. In the second part, the life-form-interview was used to elicit experiential knowledge (Haavind, 2011). This approach implies that the interviewer invites the participants to describe the day before in detail. The interviewer encouraged the participants to describe what they did, how they experienced the situation, and whether this was a typical everyday activity. In order to obtain nuanced descriptions grounded in everyday life situations, the interviewer also asked about concrete experiences of GD, such as how they handled any related distress (Haavind, 2011). In this way, the life-form interview is suited to uncover subjective experiences of GD in daily life that are taken for granted, because our being-in-the-world is more experiential than cognitive (Vetlesen & Stänicke, 1999). Each interview lasted approximately 1.5 hours.

### Procedure

The interviews were conducted and transcribed by the first author. In addition to his education as clinical psychologist, the first author has been engaged in LGBTQ activism. The first author did not work in the national treatment service for gender incongruence. Before the study’s interviews began, a pilot interview was conducted to test the suggested interview guide and make necessary adjustments. The interviewer made field notes during all the interviews, which were used as background information during the analysis. The interview guide was revised five times throughout the process, based on input from the participants. In addition, the reference group (consisting of representatives from three patient and LGBTQ organizations) was informed about the progress at three occasions during the data collection and gave feedback on interview technique. The feedback focused on what questions and formulations could be helpful in making the participants feel safe and encouraging reflection during the interview.

The participants received a written request about participation in advance of a clinical appointment and contacted the first author if they wanted to participate. In total, we sent a letter to 40 newly referred patients, and 15 of them contacted the interviewer. The interviews were conducted between December 2018 and June 2019. Eight of the interviews were conducted outside of the hospital, in the participant’s local environment (e.g., public library, school), and the remaining seven were conducted in relation to a clinical appointment.

The current study was approved by the regional committee for medical research ethics. Measures were taken before, during, and after the interviews to ensure that the participant’s integrity and autonomy were looked after, especially since the participants belonged to a vulnerable group as patients receiving healthcare (Kvale & Brinkmann, 2009). The interviewer made it clear that the participants could withdraw at any time, and they were reminded about this opportunity throughout the interview. They received health care at the national treatment unit regardless of whether they decided to participate in research. For participants under 16 years of age, permission to participate was also obtained from the parents. Before the recruitment process started, routines were established in order to make sure that participants would be followed up by their clinician, in case the research interview elicited traumatic or powerful emotional responses. Furthermore, the interviewer was a clinical psychologist and therefore competent to continuously evaluate the participants’ reactions throughout the interview.

### Analysis

As a first step in the analytic process, the first author wrote a narrative for each participant that summarized the biographical data and the most important experiential topics from the life-form-interview (Haavind, 2011). The aim of the narrative reports was to ensure that the individual complexity and variety in how subjective experiences of GD unfold were maintained during the identification of patterns across participants (Willig, 2008).

In order to grasp the breadth of the material, we used thematic analysis to identify patterns within the data. We followed the guidelines for thematic analysis outlined by Braun and Clarke (2006) in order to ensure a “bottom-up” approach. In line with a phenomenological approach, an important goal with the analysis was to be open for new and surprising findings about GD (Smith et al., 2009). The first author started to read the interviews with as open a mind as possible, making notes in the margins, a process corresponding to the first phases of thematic analysis: (1) Familiarizing yourself with your data, and (2) Generating initial codes. The writing-up of the narrative reports was also an important part of these two phases. The authors as a group discussed the initial list of codes and searched for potential themes, corresponding to the third phase suggested by Braun and Clarke (2006, p. 87): (3) Searching for themes. In this way, the first author was continuously discussing the analytic process in order to reflect on decisions and future progress. In addition, the reference group was also consulted in each round, being presented short descriptions of each potential theme, in line with the fourth phase suggested by Braun and Clarke (2006, p. 91): (4) Reviewing major themes. The potential themes were refined four times, from an initial list of 32, and reduced to the five major themes that form the basis of the findings presented in the result section. This process was helped by the use of NVivo software. In the end, the quotes were translated into English by the first author and revised by the other authors. The quotes were slightly revised in order to improve the readability, in accordance with Kvale and Brinkmann (2009).

## Results

In the following, we describe the five major themes that emerged in our interviews with TGNC adolescents experiencing GD. The first major theme, (1) Bodily sensations, serves as the principal topic of the analysis, since descriptions of the body were connected to the five remaining major themes: (2) Emotional memories from the past, (3) The process of coming-out, (4) Understanding oneself through others, and (5) Negotiating GD in everyday life. The major themes refer to different categories of experiences that together constitute GD. While we treat the major themes separately in the result section, in real life, the themes are connected and overlapping. We consider how the major themes are related in the phenomenological analysis in the discussion. To elucidate each major theme, we have provided a selection of illustrative quotes, with the participant’s age and pseudonym given in parenthesis (Table 1).

**Table 1:** Major themes with illustrative quotes

*First Major Theme: Bodily Sensations* Bodily sensations refer to various forms of experiences of the body that emerge regularly throughout the day. Certain body parts are especially distressful, and the experiences of the body do often have a sensory and tactile quality. These experiences of the body serve as constant reminders of GD throughout the day, with some contexts being worse than others. Bodily sensations are about examining oneself and dealing with the emotions it brings up	*Illustrative quotes demonstrating major theme* “The upper part is what’s troubling me, breasts and such things. I don’t use binder either, because it’s not possible to hide, so I just feel worse, it’s a reminder that you have boobs”. (Adrian, 18) “When it comes to showering it is very, very uncomfortable, because I have to sort of touch the parts I hate the most with myself, to clean myself in the shower”. (Noah, 18 years) “In school I don’t think much about it actually. When we are sitting at the desk, I use to have the chair quite low … since I am pretty tall, the chair tends to be a little short, and sometimes with a jacket, since we use to have the window open, then I don’t think much about it [gender dysphoria]”. (Oscar, 14 years)
*Second Major Theme: Emotional Memories from the Past* In addition to the body, emotional memories from the past emerged as essential when the participants described their subjective experiences of GD. The participants described emotional memories of feeling different and left outside among peers in childhood. For some, this feeling of being different was related to gender, while for others it was a more global feeling of not belonging. In addition, all participants experienced the onset of puberty as distressing, because the body changed. For many of the participants, the onset of puberty created an almost traumatic memory that still haunts them today. Together, these distressing memories are reactivated in present time and contribute to GD	*Illustrative quotes demonstrating major theme* “It was a difficult childhood, to be honest. I didn’t know where I should be, if I should be with boys or girls, so I ended up in the middle, and then I had to join the girls, but I didn’t feel quit welcome there”. (Jonas, 16 years) “Ever since I was little, I have not been normal. I have always been the one that was different”. (Ulrik, 16 years) “I have always been very boyish in school and used boyish clothes and such things, and then suddenly my body did not fit into the same clothes and I became different from the boys. That was a thing that I really, really did not like [with puberty]”. (Oscar, 14 years)
*Third Major Theme: The Process of Coming Out* At one point, all participants have been introduced to knowledge about TGNC, gender diversity or gender-affirmative healthcare aimed at changing the body. Together with the participants’ distressful relation to their body and the emotional memories of being different from peers, this introduction to TGNC-related topics has resulted in a gradually increasing mismatch between their gender identity and assigned sex at birth. Over time, the gradually increasing mismatch has resulted in a process of coming out as TGNC that consequently has transformed the participants’ understanding of their body and their past	*Illustrative quotes demonstrating major theme* I had kind of not thought like that before, exactly, that I had to be something else than a woman, because it had kind of never struck me as an opportunity”. (Noah, 18 years) One day … I walked into the bathroom, and the term “born in the wrong body” came back to me, and then it was like, “Yes, that’s what I am; I am born in the wrong body.” (Benjamin, 16 years) “When I look back now, I can clearly see that things I have done since I was very little, I now understand [I did these things] because I was trans”. (Oscar, 14 years)
*Fourth Major Theme: Understanding Oneself through Others* Interaction with other people turned out to be pivotal when the participants described subjective experiences of GD in present everyday life. When the participants interact with other people, subjective experiences of GD tend to increase, because they are afraid of being revealed as TGNC, or compare themselves with other (cisgender) men. However, forming relations with other people can also help participants relate to their body in new ways that decrease subjective experiences of GD. Over time, interactions with other people have contributed to how the participants identify today	*Illustrative quotes demonstrating major theme* There were two extremely buff guys there, so I felt extremely small. Sometimes I make that mistake—I compare myself with others, so it becomes more uncomfortable. They were maybe just five centimeter taller than me. They were just much more muscular then me”. (Alexander, 17 years) “She says, ‘It doesn’t look like you have a girl’s body at all. I see that you are a boy.’ That’s good for me to hear”. (Roy, 18 years)
*Fifth Major Theme: Negotiating GD in Everyday Life* Nowadays, the participants take measures in their everyday life to feel whole and complete, and they have succeeded to a certain degree in decreasing subjective experiences of GD. Furthermore, all participants, except from Ella, have committed to a male identity and strive to be seen as “ordinary” men. However, many participants continue to experience subjective experiences of GD. As a consequence, some feel ashamed and guilty about not overcoming GD in everyday life	*Illustrative quotes demonstrating major theme* “It feels good … it feels extremely right [to be treated as a man]”. (Jonas, 16 years) “I am sure the trans community is an okay community, and Pride and all that, but I don’t feel that I am… one of them, if you see what I mean, I don’t want to show that I am transsexual, I just want to be a normal man”. (Casper, 16 years) “Then the thoughts started to come. It was like, ‘You run too feminine’, and ‘everything about you and your personality is too feminine’. And, ‘Even if you wear a binder, you don’t look like a boy, because you still have hips that are too wide’. Suddenly, all these thoughts just came into my head when I was going to run quietly for a couple of minutes, and then I ran to the bathroom and just sat there and cried for ten minutes”. (Noah, 18 years) “If something goes wrong, I think that it’s my fault, even if it’s not, so I just think that maybe it’s my fault that I am [born in the wrong body], but I know that’s wrong, when I think about the situation then all kind of thoughts are coming and in the end I feel that it is all my fault that I am born in the wrong body”. (Adam, 14 years)

### Major Theme 1: Bodily Sensations

Bodily sensations refer to various forms of experiences of the body that emerge regularly throughout the day. Certain body parts are especially distressful, and the experiences of the body do often have a sensory and tactile quality. These experiences of the body serve as constant reminders of GD throughout the day, with some contexts being worse than others. Bodily sensations are about examining oneself and dealing with the emotions it brings up.

For some participants, the lower parts of the body—for example, the vagina, the thighs or the hips—are experienced as more distressful. For others, it can be the upper parts—for example, the breasts or the shoulders: “The upper part is what’s troubling me, breasts and such things. I don’t use binder either, because it’s not possible to hide, so I just feel worse, it’s a reminder that you have boobs” (Adrian, 18 years). Adrian considers his breasts to be large compared to peers at the same age, and this is experienced as a constant reminder of his bodily reality. Casper shares a similar experience. One of the first things Casper do when he leaves bed in the morning is to look into the mirror: “I do it every morning. I look at myself in the mirror and I think ‘ugh, what the fuck.’ And then I put on the binder, [the breasts] get more flat, but not as flat as I wish they were” (Casper, 16 years). Thus, the sensations of the body seem to be momentarily dominating when the participants become aware of it. Other typical parts of the body that were noted to create subjective experiences of GD are the hips, the feet, the thighs, the shoulders, and the face.

Jonas describes a typical bodily sensation when he looks into the mirror and sees his body: “I can look straight into my eyes, but I try not to look at the rest of the body, because then I just get uncomfortable, and I have to look away immediately… I get goosebumps all over my body” (Jonas, 16 years). Here, Jonas describes a feeling that is almost without words, but at the same time very certain and informative of how he feels. Noah is mostly able to cope with his body throughout the day. However, he becomes aware of the body when he has to shower: “When it comes to showering it is very, very uncomfortable, because I have to sort of touch the parts I hate the most with myself, to clean myself in the shower” (Noah, 18 years). Thus, the body emerges through different modalities in relation to daily activities. It seems that the act of touching the body forces Noah to relate to thoughts and feelings that he otherwise evades.

Furthermore, one’s awareness of the body changes throughout the day depending on the context. Because of the facilities at school, Oscar is able to sit in a certain position that hides his breasts:

In school I don’t think much about it actually. When we are sitting at the desk, I use to have the chair quite low … since I am pretty tall, the chair tends to be a little short, and sometimes with a jacket, since we use to have the window open, then I don’t think much about it [gender dysphoria]. (Oscar, 14 years)

He uses the cool temperature in the classroom as an excuse to wear a jacket for more coverage, almost as a way of disciplining the body to align with norms regarding the male body. This makes it possible for him to forget his body. However, Oscar is not able to ignore his beasts without fail: “In school, during break, if I feel that it starts becoming loose, and then I feel bad about myself” (Oscar, 14 years). Thus, Oscar has developed strategies to evade the body in certain situations, until the context changes and he becomes aware of the body. As previously mentioned, the mirror is one of the most frequent places where the participants are confronted with their body and become aware of it. Gabriel feels comfortable looking at himself in the mirror as long as he wears clothes. If he is naked, on the other hand, the subjective experiences of GD increase: “It feels a bit strange. One thinks that one should have looked different. It just feels like one has the wrong body. It’s just [the body] that is wrong” (Gabriel, 16 years). Thus, for many of the participants, the mismatch between how the body looks and how they feel about themselves become present when they look into the mirror.

To summarize, all participants shared a sense of not feeling comfortable in their body. The body elicits gender dysphoric experiences throughout the day.

### Major Theme 2: Emotional Memories from the Past

In addition to the body, emotional memories from the past emerged as essential when the participants described their subjective experiences of GD. The participants described emotional memories of feeling different and left outside among peers in childhood. For some, this feeling of being different was related to gender, while for others it was a more global feeling of not belonging. In addition, all participants experienced the onset of puberty as distressing, because the body changed. For many of the participants, the onset of puberty created an almost traumatic memory that still haunts them today. Together, these distressing memories are reactivated in present time and contribute to GD.

In regards to feeling like an outsider, some participants experienced being perceived as boys or very masculine girls during childhood:

It was a bit challenging in the beginning in first grade [in primary school], because everyone that was older than me thought it was a bit strange [that] I was not like all the others. All the others had a name [that matched] their gender, but when I said that my name was Ella, people thought my name was Erik, so they didn’t understand, and then they looked at me as if I was a weirdo. (Ella, 13 years)

Throughout her childhood, Ella was perceived as a boy, because of her short hair and baggy clothes. Ella experienced that others were surprised when it turned out that she had a female name. As a consequence, Ella felt that she did not belong to either the boys or the girls.

Jonas had a similar experience, saying that he remembers vividly that he did not know where to go when girls and boys were separated in school:

It was a difficult childhood, to be honest. I didn’t know where I should be, if I should be with boys or girls, so I ended up in the middle, and then I had to join the girls, but I didn’t feel quit welcome there. (Jonas, 16 years)

This emotional memory of not fitting in among the children contributes to subjective experiences of GD in present time.

Other participants described a vague feeling of being ostracized from peers, but it was not necessarily related to gender: “Ever since I was little, I have not been normal. I have always been the one that was different” (Ulrik, 16 years). Ulrik struggled to find a community with peers. However, he explained that he was not uncomfortable with being a girl in childhood. It seems that this strong memory of being alienated from peers contributes to subjective experiences of GD today. Thus, whether or not the experience of fitting in was related to gender, all participants carry with them emotional and distressful memories of a struggling to find a place among peers.

Finally, all participants experienced the onset of puberty as extremely stressful. For some, the onset of puberty confirmed a longtime concern about not being a girl. For others, the onset of puberty prompted questioning about gender identity for the first time. Oscar had never considered himself to be male during childhood, but he remembers that he was a “tomboy” and allowed to present in a more “boyish” manner. However, he experienced that the onset of puberty changed his social role:

I have always been very boyish in school and used boyish clothes and such things, and then suddenly my body did not fit into the same clothes and I became different from the boys. That was a thing that I really, really did not like [with puberty]. (Oscar, 14 years)

In this quote, Oscar describes how the bodily development during puberty made it impossible to wear boyish clothes anymore. Furthermore, it seems that puberty left some participants with perhaps a traumatic memory of not being in control of the development of the body.

Thus, the participants shared an experience of entering adolescence with strong emotional memories of being different from peers. For some, this sense has always been related to gender. For others, they did not start to question their gender identity until after puberty onset. However, all participants are haunted by these emotional memories of today.

### Major Theme 3: The Process of Coming Out

At one point, all participants have been introduced to knowledge about TGNC, gender diversity, or gender-affirmative healthcare aimed at changing the body. Together with the participants’ distressful relation to their body and the emotional memories of being different from peers, this introduction to TGNC-related topics has resulted in a gradually increasing mismatch between their gender identity and assigned sex at birth. Over time, the gradually increasing mismatch has resulted in a process of coming out as TGNC that consequently has transformed the participants’ understanding of their body and their past.

For many of the participants, the process of coming out started when they were introduced to knowledge about TGNC, gender diversity or gender affirmative care. Reported sources of knowledge included books, movies, and social media, as well as peers who had come out as transgender. Also, some participants give credit to family members who suggested the possibility of seeking gender affirmative care. In Adam’s case, he was perceived to be a “boyish” girl during childhood, because he never wanted to be a girl. However, something changed when he learned that a distant family member started on medical treatment in order to change gender:

I have never understood why I have felt different from others, but when my second cousin came out, I understood that it was something with me as well, because I understood that it was an alternative [to change gender]. This knowledge introduced new words, it made me much happier, because I knew that it was an opportunity to change my confusing situation. (Adam, 14 years)

It seems that learning about the opportunity to change gender was a transformative experience for Adam, because he understood that he had “not been complete” before. Noah shares a similar experience: “I had kind of not thought like that before, exactly, that I had to be something else than a woman, because it had kind of never struck me as an opportunity” (Noah, 18 years). Thus, it seems that for both Adam and Noah, the awareness of being TGNC was a great relief, because it offered an explanation for why they felt different from peers.

The process of coming out was described by the participants as gradual and dynamic. Initially, Noah came out as non-binary, or “without gender,” as he describes it. However, he experienced that people around him did not understand his new gender identity:

For me, being without gender was kind of like, “okay, without gender, that’s okay, it’s a good place to be.” I felt that at least [other people] wanted to accept it, but everyone still thought I was a girl … so I felt more and more masculine, I felt that being without gender was perhaps not right anymore, so then I figured out that I was a boy. (Noah, 18 years)

Thus, it seems that for many participants the process of coming out was characterized by movements back and forth, depending on the reactions from others. Noah, for example, started out as non-binary but ended up identifying as male, because he felt that other people did not understand him. Furthermore, it seems that the mere knowledge of other TGNC people and the opportunity to seek medical treatment made many participants start questioning their gender identity. This culminated in one day suddenly realizing that transgenderism was a reality for them. Benjamin had heard about someone being “born in the wrong body” some months before he realized that this was relevant for himself:

One day … I walked into the bathroom, and the term “born in the wrong body” came back to me, and then it was like, “Yes, that’s what I am; I am born in the wrong body.” (Benjamin, 16 years)

The acknowledgement of being TGNC could be sudden, following a period of time when one had started learning about transsexualism or transgenderism. With this knowledge, the participants report gradually beginning to reflect on their past in a new light, until they one day realized that this was relevant for them.

After having gone through the process of coming out, many of the participants started to understand their past differently: “When I look back now, I can clearly see that things I have done since I was very little, I now understand [I did these things] because I was trans” (Oscar, 14 years). Thus, for Oscar, it seems that new knowledge about TGNC and medical treatment to change gender identity offered a new framework to understand himself, his relation to the body and his emotional memories of being different from peers.

To summarize, it seems that the process of coming out has been a transformative experience that has changed how participants understand their past. The process of coming out has been a relief for many participants, because it has provided a meaningful framework to understand their emotional memories of being different and outside.

### Major Theme 4: Understanding Oneself Through Others

Interaction with other people turned out to be pivotal when the participants described subjective experiences of GD in present everyday life. When the participants interact with other people, subjective experiences of GD both decrease and increase, depending on the context.

Many of the participants experience that GD increases when they are in public spaces, such as school, because they are concerned about being revealed as TGNC:

Before lunch it’s usually a couple of visits to the toilet, and I always have to be careful if I use paper that the sound must not be too loud, because I don’t want people to hear that I pee and have to use paper. (Benjamin, 16 years)

Thus, when Benjamin visits the public toilet in school, he is concerned that other people disclose his background as girl. These concerns contribute to subjective experiences of GD. Furthermore, subjective experiences of GD tend to increase when the participants find themselves in a situation where they compare themselves with other (cisgender) men. Alexander was in the training studio when two men were suddenly standing beside him:

There were two extremely buff guys there, so I felt extremely small. Sometimes I make that mistake—I compare myself with others, so it becomes more uncomfortable. They were maybe just five centimeter taller than me. They were just much more muscular then me. (Alexander, 17 years)

The men made Alexander feel small, and hence less male. His experience is a salient example of how GD can be relational and depend upon context. Other contextual factors that trigger subjective experiences of GD among the participants are the growing social differences between boys and girls in adolescence that force the participants to choose more explicitly between the social gender roles.

Forming romantic or friendly relations with peers as part of entering adolescence could both increase or decrease subjective experiences of GD. Roy experienced that he was better able to cope with GD after he met his first girlfriend:

Her friends and everyone were gathered, and she took the risk of asking me, “Are you born in the wrong body?” I started to laugh, and then I said, “Yes. Why do you ask?” She said just one word, “Cool.” Then, “That’s fascinating. Could you tell me more?” (Roy, 18 years)

After this, Roy was able to form a long-term relationship with his girlfriend, Jeanette. When Roy and Jeanette have sex, they both acknowledge that his genitals are “female.” Jeanette identifies as heterosexual but has come to terms with the relationship by defining Roy as a man in a woman’s body: “She says, ‘It doesn’t look like you have a girl’s body at all. I see that you are a boy.’ That’s good for me to hear” (Roy, 18 years). Roy does not like to look at himself in the mirror when alone. However, when he is with Jeanette, his body becomes a more comfortable place to be. It seems that the relation to Jeanette has been a transformative experience for Roy and how he perceives his body. This process has significantly decreased his subjective experiences of GD.

To summarize, subjective experiences of GD are influenced by interaction with other people. Most participants experience to various degrees that subjective experience of GD increase when they are together with other people. However, forming relations with other people can also help participants relate to their body in new ways that decrease subjective experiences of GD. Over time, interactions with other people have contributed to how the participants identify today, which leads us to the fifth major theme.

### Major Theme 5: Negotiating Gender Dysphoria in Everyday Life

Nowadays, the participants take measures in their everyday life to feel whole and complete, and they have succeeded to a certain degree in decreasing subjective experiences of GD. Furthermore, all participants, except from Ella, have committed to a male identity and strive to be seen as “ordinary” men. However, many participants continue to experience subjective experiences of GD. As a consequence, some feel ashamed and guilty about not overcoming GD in everyday life.

Participants struggle to feel whole and complete, in order to decrease subjective experiences of GD. When the participants are addressed by others as men, they feel whole and complete. Adam feels good when someone says uses the male pronoun “he” about him: “It simply feels right, and it feels right to answer back [when they say ‘he’]” (Adam, 14 years). It seems that being addressed as a man makes it easier for Adam to react to the requests from others. Jonas describes a similar feeling when other people treat him as a man: “It feels good … it feels extremely right [to be treated as a man]” (Jonas, 16 years). Similarly, when Noah and his boyfriend are taken to be a gay couple among friends, he feels more comfortable: “With people we know, who know I am a boy and so see us as a gay couple, it’s no problem” (Noah, 18 years). As a consequence of this struggle to unify inner gender identity with external expression, all participants, except for Ella, have committed to a male identity. Some describe themselves as “being born in the wrong body” while others articulate a belonging to the “transgender movement”. Some participants, such as Jonas, have felt a strong affinity to typically masculine qualities and interests since early childhood. Other participants, such as Noah, have only recently started identifying with men.

A parallel experience to feeling whole and complete is a longing among many of the participants to “just be yourself” and a “normal guy”. Roy describes that he often forgets his body throughout the day, probably because he mostly passes as a man. However, there are times when he remembers: “Every time I shower, I look at my body in the mirror, and I see there is something wrong with it. I don’t like it. I wish I were a boy—a real boy, born like a boy” (Roy, 18 years). This quote indicates that Roy carries with him a deep longing to have a “real” male body, despite the fact that he usually passes as a man. Casper feels uncomfortable about being associated with what he refers to as the “trans community,” because he wants to be a “normal guy”:

I am sure the trans community is an okay community, and Pride and all that, but I don’t feel that I am… one of them, if you see what I mean, I don’t want to show that I am transsexual, I just want to be a normal man. (Casper, 16 years)

It seems that Casper aims to reach a state where he is “just a normal person,” without the sense of appearing exaggerated and unnatural.

However, the identification as men and the process of coming-out and feeling whole has for many participants come at a price—they ruminate more on gender and their body than before. Noah, for example, describes a typical experience when he was exercising:

Then the thoughts started to come. It was like, “You run too feminine,” and “everything about you and your personality is too feminine.” And, “Even if you wear a binder, you don’t look like a boy, because you still have hips that are too wide.” Suddenly, all these thoughts just came into my head when I was going to run quietly for a couple of minutes, and then I ran to the bathroom and just sat there and cried for ten minutes. (Noah, 18 years)

Noah struggles with ruminations about his body and gender expression that were not problematic before he committed to a male identity. Benjamin describes a similar experience:

Now I get depressed when I think about being born a girl, or I blame myself for everything, just the fact that I can’t have my own children [due to masculinizing hormone treatment], I can cry an entire afternoon because I have shoe size 38, I have small feet, such ridiculous stuff … I have sort of this demon within me. (Benjamin, 16 years)

For some participants, such as Noah and Benjamin, they tend to dislike their body even more than before they came out as transgender and started to identify as men. It seems that the new identification as male also tends to increase subjective experiences of GD.

Furthermore, many participants tend to feel ashamed about experiencing GD:

If something goes wrong, I think that it’s my fault, even if it’s not, so I just think that maybe it’s my fault that I am [born in the wrong body], but I know that’s wrong, when I think about the situation then all kind of thoughts are coming and in the end I feel that it is all my fault that I am born in the wrong body. (Adam, 14 years)

Therefore, it seems that Adam tends to feel guilty about experiencing GD. Gabriel, who’s not yet completely open about being a transgender man in high school, says that pupils in his school are conservative, and he often wishes that he were born as “just a normal boy.” When he encounters gay and lesbian youth in the classroom, he feels ashamed, explicitly stating that he does not want to be associated with “anything queer.”

Every time they [a group of gay and lesbian classmates] enter the classroom, they typically walk around and talk about gender identity in a very hysterical and exaggerated style, they color their hair, I think they are so weird. (Gabriel, 16 years)

Gabriel, like many other participants, is afraid of discrimination in school. Instead of attributing the associated distress to societal factors, it seems that many participants have a tendency to direct the negative feelings toward themselves. Thus, like Adam, it seems that Gabriel tends to internalize the stress he experiences and blame himself.

To summarize, the fifth major theme indicates a complex negotiation of GD in everyday life. The participants are striving to reach a state of feeling whole, where they can “just be themselves.” This has led to a commitment to identify as men for all participants, except from Ella. Furthermore, this commitment to a male identity has transformed the participants’ relations to their own bodies. On the one hand, this identification has made the participants feel whole and complete. On the other hand, this has come at a price, because some participants seem to have developed new forms of GD.

## Discussion

The present study explored which subjective experiences that adolescents assigned female at birth target as essential when interviewed about GD in their daily life. The first major theme indicates that the body triggers subjective experiences of GD throughout the day. The second major theme suggests that emotional memories of being different from peers emerged as significant when participants describe their subjective experiences of GD. For some, this feeling of being different is related to gender, while for others it is a more global feeling of not belonging. The third major theme indicates that all participants have been introduced at some point to knowledge about TGNC, gender diversity or gender-affirmative healthcare. Together with the participants’ distressful relations to their body and the emotional memories of being ostracized from peers, this has resulted in a gradually increasing mismatch between their gender identity and assigned sex at birth, and consequently the process of coming out as TGNC. This process has transformed the participants’ understanding of their body and their past. The fourth major theme describes how subjective experiences of GD both increase and decrease when participants are with others. Finally, the fifth major theme suggests a complicated negotiation in everyday life. One the one hand, the participants are striving to reach a state of feeling whole by presenting as “ordinary” men. On the other hand, this has come at a price, because some participants have developed new forms of GD as a consequence of committing to a male identity. Today, many participants tend to dislike their body even more than before they started to identify as men.

Interestingly, the importance of the body, the onset of puberty, psychosocial changes associated with adolescence, and the impact from forming relations to peers coincide with the topics reported in previous qualitative studies on gender identity development of TGNC youth with GD (Catalpa & McGuire, 2018; McGuire et al., 2016; Pollock and Eyre, 2010; Steensma et al., 2010; Wilson et al., 2005). These challenges are typical difficulties among adolescents generally (Kaltiala et al., 2018), and among TGNC youth with GD especially (Kaltiala et al., 2020b; Leibowitz & de Vries, 2016; Olson-Kennedy et al., 2016; Zucker, 2019). Furthermore, the current body of knowledge indicates that TGNC youth suffering from GD is a heterogeneous group with various clinical needs (Janssen et al., 2019). Some youth continue to struggle with co-occurring mental health challenges after gender-affirmative medical care, while others seem to benefit from the treatment (Carmichael et al., 2021; Kaltiala et al., 2020b). The findings from the present study indicate that the process of negotiating subjective experiences of GD requires an ability to form relations with other people that enable new ways of doing gender in everyday life. This resonates with qualitative research on gender affirmation among TGNC adults regarding the necessity to enact preferred gender roles in order to be recognized by others (Loza et al., 2017). Thus, being able to handle GD in everyday life requires interpersonal skills in order to form vital relations to other people. Perhaps TGNC youth who seem not to profit from gender-affirmative medical care are struggling with these psychosocial tasks in adolescence and the entrance into adulthood (Kaltiala-Heino et al., 2018). Furthermore, perhaps the participants are struggling with what has been referred to as the third phase in TGNC gender identity development (exploration of gender expression), and the fourth stage (connecting to others) (Bockting, 2014). The results from the current study suggest that GD is a complex social and bodily phenomenon that continuously has to be negotiated. Having this complexity in mind, perhaps it should be no surprise that the current body of knowledge indicates that some TGNC youth profit from medical treatment, while others continue to struggle with co-occurring mental health challenges.

Furthermore, the results from the current study suggest that the participants are facing a dilemma when negotiating GD. The participants share a common experience of having felt different from others in childhood, which might have led to an increasing identification as men during the process of coming out. On the one hand, they feel more complete and whole after having embraced a male identity. This is understandable, given the importance of belonging to a recognized social group (Segal, 2008). On the other hand, this process of identification seems to have created a longing to be “ordinary” men. However, this longing does not match the participants’ bodies and their background as assigned female at birth. It seems that the awareness of gender-affirmative care and the process of coming out as TGNC has brought with it new standards for what one is feeling and how to deal with these feelings, so it puts pressure on the participants to attain certain goals, such as living as “ordinary men”. This dilemma is open for a queer reading: The norms governing how men should behave and male bodies should be are strict in a heteronormative society (Roen, 2016). In order to be comprehensible, the participants seem to have gradually started identifying as men. As a consequence, they are to various degrees recognized as men today, and they feel more complete than before. At the same time, this new identification has transformed their relation to their body, and they compare themselves with other (cisgender) men. In this competition, most of the participants end up feeling less male. It seems that this leads many participants to feel ashamed and guilty. They moreover tend to dislike their body even more than before they started to identify as men and came out as transgender. The participants are not able to fulfill traditional gender norms regarding body and identity. They continue to struggle at the margins of gender—a dilemma that has been referred to as a “queer failure” on behalf of youth (Roen, 2016). Furthermore, perhaps the increased prevalence of co-occurring mental health challenges among TGNC youth is a symptom of this queer failure. In that vein, the present study indicates a more complex process of negotiating GD than has been described in the current body of knowledge on TGNC adolescents. How can this complex negotiation of GD be conceptualized?

### Toward a Conceptual Model of Gender Dysphoria

In order to better understand how subjective experiences of GD arise and unfold, we suggest a conceptual model that specifies how the five major themes that emerged from the analysis are related. The first two major themes—(1) Bodily sensations and (2) Emotional memories from the past—can be conceptualized as sources that elicit subjective experiences of GD. These sources are not meaningful in themselves, but must be mediated through psychological operations, consisting of the next two major themes—(3) The process of coming out, and (4) Understanding oneself through others. The present study indicates that subjective experiences of GD arise in and through the transformative process of coming out as TGNC, and the continuous understanding of oneself in relation to other people. This process results in an ongoing state of negotiating GD, as described in the final major theme, (5) Negotiating GD in everyday life. Thus, we suggest that subjective experiences of GD are the result of a continuous process of sources being mediated through psychological operations into states.

**Fig. 1:**
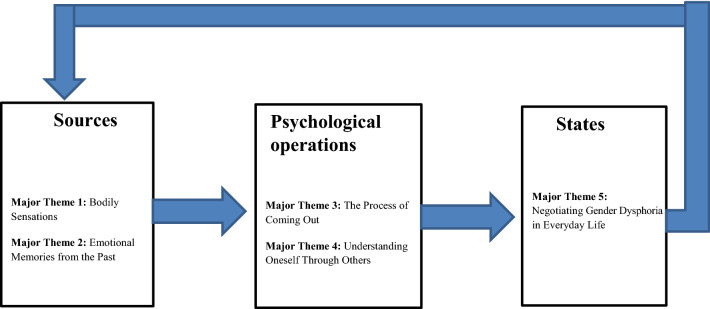
Outline of the Conceptual Model of Subjective Experiences of Gender Dysphoria

Furthermore, we suggest that a certain state, for example, the identification as a man, influences how the participants feel about their body. Consequently, this leads to a new understanding of the body, i.e., that the body is perceived to be too feminine. The consequence of this new state is that the body emerges as a source that elicits new subjective experiences of GD, described in the fifth major theme as increased rumination about one’s body and feelings of shame. The result is a constant interplay between sources being mediated into states through psychological operations, resulting in a continuous production of subjective experiences of GD that are situated in everyday life (see Fig. 1). In this way, the model might account for the development of new forms of subjective experiences of GD over time. Translated to the present study, bodily sensations that make the participants uncomfortable as well as emotional memories of pre-adult ostracization are sources that are mediated through psychological operations, such as coming-out, committing to a male gender identity, and understanding oneself through others. This results in different states: Participants report that subjective experiences decrease in certain situations and increase in others. The dynamic between sources, psychological operations, and states might shed light on the experiential aspects underlying developmental models of TGNC identity and transition between the stages. Furthermore, the conceptual model of subjective experiences of GD could ideally contribute with a phenomenological background to researchers that are interpreting data on TGNC youth and livelihoods.

### Clinical Implications

Moreover, we suggest that this model may offer guidance for clinicians, namely a strategy to engage TGNC youth in a psychological treatment process of exploring and reflecting on their subjective experiences of GD (Butler et al., 2018). Ideally, such a process could help both clients and clinicians discuss the need for medical treatment alongside identity exploration in general (Zucker, 2019). This can be especially relevant for those TGNC youth who struggle with co-occurring psychopathology (Carmichael et al., 2021; Kaltiala et al., 2020b). Thus, the development of the model is an attempt to go beyond the oft-polarized discussion among clinicians and academics regarding the relation between psychopathology and GD in order to assess the unique pathways of development and maintenance of each youth (Meyer-Bahlburg, 2019). Firstly, the framework of sources, psychological operations, and states that together create unique dynamics of subjective experiences of GD can serve as the starting point of the clinical assessment in the individual TGNC youth. Clinicians may start by asking their clients about sources that contribute to their subjective experiences of GD, such as how they experience their body and how childhood and the onset of puberty have been. Then, we encourage clinicians to ask their clients about experiences with coming out and how they relate to other people that influence their subjective experiences of GD throughout the day, in order to get insight into typical psychological operations. In addition, it can be helpful to ask clients about their states—how they identify and what their feelings are in different situations. The aim of this process is to get an overview of how clients understand themselves, how they have related to their bodies throughout childhood, and how subjective experiences of GD have developed over the years.

Secondly, the model can serve as an entry into exploring how TGNC youth experience GD in their individual ways throughout the day, especially by paying attention to rumination and typical train of thoughts that increase subjective experiences of GD. This can be helpful in order to assist clients in an exploration of the unique dynamic of sources, psychological operations, and states that maintain their subjective experiences of GD and create suffering in daily life. Assisting young clients in a reflective process around influential lived experiences and how they relate to the body, as well as important relations to peers and family, can be helpful for all TGNC youth regardless of whether they end up seeking medical care. Ideally, the model can be used to break down into smaller pieces the oft-overwhelming subjective experiences of GD that can be difficult to describe with words. The clinical focus should always be on the idiosyncratic dynamic of GD of each young TGNC individual and their wider social context (Bell, 2020; Saketopoulou, 2020; Wren, 2019).

### Limitations

There are three limitations that pose a challenge to the current study. Firstly, all participants were recruited from a clinical help-seeking population. The clinical background could perhaps have influenced the results, especially the challenges they are experiencing in relation to self and others, and emotional memories from the past. Perhaps TGNC adolescents who do not seek medical treatment experience less challenges in relation to their non-conforming gender identity. Secondly, although we aimed to recruit participants early in their treatment process, six of the youth had already started on medical treatment prescribed by other health personnel. This could potentially have influenced their subjective experiences of GD, because bodily alterations might influence on their gender identity and how they relate to other people. Thirdly, we excluded participants with psychosis and suicidal behavior. This might have consequences for the generalizability of the sample; perhaps the adolescents interviewed in the current study represent a more proactive and reflected group of young people with GD.

### Future Research

The present study indicates that subjective experiences of GD are the result of a complex interplay between sources, psychological operations, and states. Ideally, the model suggested in the present study could contribute to an improved conceptual understanding of GD that incorporates current scientific knowledge on TGNC youth across disciplines. Furthermore, future qualitative studies should explore subjective experiences of GD among TGNC youth who have undergone gender-affirmative medical care, especially in regard to how medical treatment has affected how they have related to important developmental milestones such as puberty and forming relations to peers. Quantitative studies are needed to identify important factors that contribute to subjective experiences of GD, such as stigma, co-occurring mental health challenges, and developmental trajectories throughout adolescence and into adulthood.

## References

[CR1] American Psychiatric Association (2013). Diagnostic and statistical manual of mental disorders.

[CR2] Arnoldussen M, Steensma TD, Popma A, van der Miesen A, Twisk JWR, de Vries AL (2019). Re-evaluation of the Dutch approach: Are recently referred transgender youth different compared to earlier referrals?. European Child & Adolescent Psychiatry.

[CR3] Austin A (2016). “There I am”: A grounded theory study of young adults navigating a transgender or gender nonconforming identity within a context of oppression and invisibility. Sex Roles.

[CR4] Bell D (2020). First do no harm. International Journal of Psychoanalysis.

[CR5] Bockting W, Tolman DL, Diamond LM, Bauermeister JA, George WH, Pfaus JG, Wards LM (2014). Transgender identity development. APA handbook of sexuality and psychology, Vol. 1. Person-based approaches.

[CR6] Bradford NS, Rider GN, Catalpa JM, Morrow QJ, Berg DR, Spencer KG, McGuire JK (2018). Creating gender: A thematic analysis of genderqueer narratives. International Journal of Transgenderism.

[CR7] Braun V, Clarke V (2006). Using thematic analysis in psychology. Qualitative Research in Psychology.

[CR8] Butler G, De Graaf N, Wren B, Carmichael P (2018). Assessment and support of children and adolescents with gender dysphoria. Archives of Disease in Childhood.

[CR9] Carmichael P, Butler G, Masic U, Cole TJ, De Stavola BL, Davidson S, Skageberg EM, Khadr S, Viner RM (2021). Short-term outcomes of pubertal suppression in a selected cohort of 12 to 15 year old young people with persistent gender dysphoria in the UK. PLoS ONE.

[CR10] Catalpa JM, McGuire JK (2018). Family boundary ambiguity among transgender youth. Family Relations.

[CR11] Chodzen G, Hidalgo MA, Chen D, Garofalo R (2019). Minority stress factors associated with depression and anxiety among transgender and gender-nonconforming youth. Journal of Adolescent Health.

[CR12] Cohen-Kettenis PT, van Goozen SHM (1997). Sex reassignment of adolescent transsexuals: A follow-up study. Journal of the American Academy of Child and Adolescent Psychiatry.

[CR13] Dahlberg K (2006). The essence of essences: The search for meaning structures in phenomenological analysis of lifeworld phenomena. International Journal of Qualitative Studies on Health and Well-being.

[CR14] de Graaf NM, Giovanardi G, Zitz C, Carmichael P (2018). Sex ratio in children and adolescents referred to the gender identity development service in the UK (2009–2016) [Letter to the Editor]. Archives of Sexual Behavior.

[CR52] de Vries ALC, Cohen-Kettenis PT (2012). Clinical management of gender dysphoria in children and adolescents: The Dutch approach. Journal of Homosexuality.

[CR15] de Vries ALC, McGuire JK, Steensma TD, Wagenaar EC, Doreleijers TA, Cohen-Kettenis PT (2014). Young adult psychological outcome after puberty suppression and gender reassignment. Pediatrics.

[CR16] Devor AH (2004). Witnessing and mirroring: A fourteen stage model of transsexual identity formation. Journal of Gay & Lesbian Psychotherapy.

[CR17] Drescher J, Cohen-Kettenis P, Winter S (2012). Minding the body: Situating gender identity diagnoses in the ICD-11. International Review of Psychiatry.

[CR18] Ehrensaft D (2017). Gender nonconforming youth: Current perspectives. Adolescent Health, Medicine and Therapeutics.

[CR19] Giovanardi G (2017). Buying time or arresting development? The dilemma of administering hormone blockers in trans children and adolescents. Porto Biomedical Journal.

[CR20] Haavind H, von der Lippe AL, Rønnestad MC (2011). Utvikling og deltagelse Livsformintervjuet som klinisk instrument med barn og unge. Det kliniske intervjuet [The Clinical Interview].

[CR21] Janssen A, Busa S, Wernick J (2019). The complexities of treatment planning for transgender youth with co-occurring severe mental illness: A literature review and case study. Archives of Sexual Behavior.

[CR22] Kaltiala R, Bergman H, Carmichael P, de Graaf NM, Rischel KE, Frisen L, Schorkopf M, Suomalainen L, Waehre A (2020). Time trends in referrals to child and adolescent gender identity services: A study in four Nordic countries and in the UK. Nordic Journal of Psychiatry.

[CR23] Kaltiala R, Heino E, Työlöjärvi M, Suomalainen L (2020). Adolescent development and psychosocial functioning after starting cross-sex hormones for gender dysphoria. Nordic Journal of Psychiatry.

[CR24] Kaltiala-Heino R, Bergman H, Työlöjärvi M, Frisen L (2018). Gender dysphoria in adolescence: Current perspectives. Adolescent Health, Medicine and Therapeutics.

[CR25] Katz-Wise SL, Budge SL, Fugate E, Flanagan K, Touloumtzis C, Rood B, Perez-Brumer A, Leibowitz S (2017). Transactional pathways of transgender identity development in transgender and gender-nonconforming youth and caregiver perspectives from the Trans Youth Family Study. International Journal of Transgenderism.

[CR26] Kvale S, Brinkmann S (2009). InterViews: Learning the craft of qualitative research interviewing.

[CR27] Leibowitz S, de Vries ALC (2016). Gender dysphoria in adolescence. International Review of Psychiatry.

[CR28] Levitt H, Ippolito MR (2014). The experience of being transgender: Navigating minority stressors and developing authentic self-presentation. Psychology of Women Quarterly.

[CR29] Loza O, Beltran O, Mangadu T (2017). A qualitative exploratory study on gender identity and the health risks and barriers to care for transgender women living in a U.S.–Mexico border city. International Journal of Transgenderism.

[CR30] McGuire JK, Doty JL, Catalpa JM, Ola C (2016). Body image in transgender young people: Findings from a qualitative, community-based study. Body Image.

[CR31] McLeod J, Lambert MJ (2013). Qualitative research: Measures and contributions. Bergin and Garfield’s handbook of psychotherapy and behavioral change.

[CR32] Meyer-Bahlburg HFL (2019). Introduction to the special section on clinical approaches to adolescents with gender dysphoria. Archives of Sexual Behavior.

[CR33] Mullen G, Moane G (2013). A qualitative exploration of transgender identity affirmation at the personal, interpersonal, and sociocultural levels. International Journal of Transgenderism.

[CR34] Olson-Kennedy J, Cohen-Kettenis PT, Kreukels BPC, Meyer-Bahlburg HFL, Garofalo R, Meyer W, Rosenthal SM (2016). Research priorities for gender nonconforming/transgender youth: Gender identity development and biopsychosocial outcomes. Current Opinion in Endocrinology & Diabetes and Obesity.

[CR35] Pollock L, Eyre SL (2010). Growth into manhood: Identity development among female-to-male transgender youth. Culture, Health & Sexuality.

[CR36] Riggs DW, Bartholomaeus C, Sansfacon AP (2019). “If they didn’t support me, I most likely wouldn’t be here”: Transgender young people and their parents negotiating medical treatment in Australia. International Journal of Transgenderism.

[CR37] Roen K (2016). The body as a site of gender-related distress: Ethical considerations for gender variant youth in clinical settings. Journal of Homosexuality.

[CR38] Saketopoulou A (2020). Thinking psychoanalytically, thinking better: Reflections on transgender. International Journal of Psychoanalysis.

[CR39] Salzburg S, Davis TS (2010). Co-authoring gender-queer youth identities: Discursive *tellings* and *retellings*. Journal of Ethnic and Cultural Diversity in Social Work.

[CR40] Segal L (2008). After Judith Butler: Identities, who needs them?. Subjectivity.

[CR41] Smith JA, Flowers P, Larkin M (2009). Interpretative phenomenological analysis. Theory, method and research.

[CR42] Steensma TD, Biemond R, de Boer F, Cohen-Kettenis PT (2010). Desisting and persisting gender dysphoria after childhood: A qualitative follow-up study. Clinical Child Psychology and Psychiatry.

[CR43] Vetlesen, A. J. & Stänicke, E. (1999). *Fra hermeneutikk til psykoanalyse. Muligheter og begrensninger i filosofiens møte med psykoanalysen* [From hermeneutics to psykoanalysis. Opportunities and limitations in the meeting between philosophy and psychoanalysis]. Oslo: Ad Notam Gyldendal.

[CR44] Willig C (2008). Introducing qualitative research in psychology.

[CR45] Wilson I, Griffin C, Wren B (2005). The interaction between young people with atypical gender identity organization and their peers. Journal of Health Psychology.

[CR46] Winter S, Diamond M, Green J, Karasic D, Reed T, Whittle S, Wylie K (2016). Transgender people: Health at the margins of society. Lancet.

[CR47] World Health Organization. (2019). *The ICD-11 classification of mental and behavioral disorders: Clinical descriptions and diagnostic guidelines*. World Health Organization.

[CR48] Wren B (2019). Reflections on ‘Thinking an ethics of gender exploration: Against delaying transition for transgender and gender variant youth’. Clinical Child Psychology & Psychiatry.

[CR49] Wylie K, Knudson G, Khan SI, Baral S, Bonierbale M, Watanyusakul S (2016). Serving transgender people: Clinical care considerations and service delivery models in transgender health. Lancet.

[CR50] Zucker KJ (2019). Adolescents with gender dysphoria: Reflections on some contemporary clinical and research issues. Archives of Sexual Behavior.

[CR51] Zucker KJ, Bradley SJ, Owen-Anderson A, Kibblewhite SJ, Cantor JM (2008). Is gender identity disorder in adolescents coming out of the closet? [Letter to the Editor]. Journal of Sex & Marital Therapy.

